# Polyglutamine Induced Misfolding of Huntingtin Exon1 is Modulated by the Flanking Sequences

**DOI:** 10.1371/journal.pcbi.1000772

**Published:** 2010-04-29

**Authors:** Vinal V. Lakhani, Feng Ding, Nikolay V. Dokholyan

**Affiliations:** Department of Biochemistry and Biophysics, University of North Carolina at Chapel Hill, School of Medicine, Chapel Hill, North Carolina, United States of America; National Cancer Institute United States of America and Tel Aviv University, Israel

## Abstract

Polyglutamine (polyQ) expansion in exon1 (XN1) of the huntingtin protein is linked to Huntington's disease. When the number of glutamines exceeds a threshold of approximately 36–40 repeats, XN1 can readily form amyloid aggregates similar to those associated with disease. Many experiments suggest that misfolding of monomeric XN1 plays an important role in the length-dependent aggregation. Elucidating the misfolding of a XN1 monomer can help determine the molecular mechanism of XN1 aggregation and potentially help develop strategies to inhibit XN1 aggregation. The flanking sequences surrounding the polyQ region can play a critical role in determining the structural rearrangement and aggregation mechanism of XN1. Few experiments have studied XN1 in its entirety, with all flanking regions. To obtain structural insights into the misfolding of XN1 toward amyloid aggregation, we perform molecular dynamics simulations on monomeric XN1 with full flanking regions, a variant missing the polyproline regions, which are hypothesized to prevent aggregation, and an isolated polyQ peptide (Q_n_). For each of these three constructs, we study glutamine repeat lengths of 23, 36, 40 and 47. We find that polyQ peptides have a positive correlation between their probability to form a β-rich misfolded state and their expansion length. We also find that the flanking regions of XN1 affect its probability to^x_page_count=28 form a β-rich state compared to the isolated polyQ. Particularly, the polyproline regions form polyproline type II helices and decrease the probability of the polyQ region to form a β-rich state. Additionally, by lengthening polyQ, the first N-terminal 17 residues are more likely to adopt a β-sheet conformation rather than an α-helix conformation. Therefore, our molecular dynamics study provides a structural insight of XN1 misfolding and elucidates the possible role of the flanking sequences in XN1 aggregation.

## Author Summary

Huntington's Disease is a neurodegenerative disorder associated with protein aggregation in neurons. The aggregates formed are thought to lead to neurotoxicity and cell death. Understanding the molecular structure of these aggregates may lead to strategies to inhibit aggregation. Exon 1 (XN1) of the huntingtin protein is critical for aggregate formation. This polypeptide has a naturally occurring polyglutamine sequence (polyQ), which is elongated in patients afflicted with the disease. The polyQ region in XN1 has several flanking sequences with distinct physicochemical properties, including the N-terminal 17 residues, two polyproline regions, and C-terminal sequences, that may affect its overall structure and aggregation. What is the overall structure of XN1, and what structural effects do the neighboring sequences have on each other and polyQ? We address these questions by studying computational models of various polypeptides, including XN1 and three mutant forms associated with Huntington's Disease. Certain neighboring sequences are found to inhibit aggregation, while others may be recruited by polyQ to form aggregates. Our results suggest the role that the flanking sequences may play in XN1 aggregation and may subsequently guide future structural models of XN1 aggregation.

## Introduction

Similar to eight other neurodegenerative polyglutamine diseases [1]–[5], Huntington's disease (HD) is associated with a polyglutamine (polyQ) expansion in the huntingtin protein. In HD, the first exon (XN1) of the huntingtin protein, which contains the polyQ region, has been implicated as the pathogenic polypeptide [6]–[9]. It is hypothesized that after it is cleaved from huntingtin, the XN1 polypeptides misfold and form lethal cellular aggregates or inclusions in neuronal cells [1]–[5]. Evidence from *in vivo* studies [6], [7] indicates that XN1 polypeptides can form aggregates similar to those observed in the neurons of afflicted patients. Despite limited knowledge on the structure and function of XN1, some information about its aggregation is known. The length of the polyQ region in XN1 is inversely proportional to the age of onset of symptoms [2], [9]. Additionally, there is a threshold of approximately 36–40 repeats, within which symptoms may or may not develop [10]. However, beyond this threshold, lethal symptoms eventually develop in the lifetime of the patient. These symptoms develop earlier in the life of patients with longer repeat lengths. *In vitro* kinetic studies of polyQ peptides have emulated this clinical correlation between age and length [11], [12]. Kinetic studies of polyQ [13] aggregation suggested that the misfolding of a polyQ monomer initiates the aggregation. Although other studies [14], [15] proposed more complex aggregation scenario, we hypothesize that the misfolding of a polyQ monomer plays a critical role in the formation of ordered amyloid aggregations. Similarly, the aggregation of XN1 displays more complicated aggregation behavior [16]–[18], [19], [20], it has been shown that the length-dependent misfolding of polyQ sequence in the XN1 monomer plays a critical role in the initial oligomerization and the later formation of β-rich amyloid aggregates [20]. Here, we propose to study the structure and dynamics of monomeric XN1, in hopes to help illuminate the aggregation mechanism and help determine the role of these aggregates.

**Figure 1:**
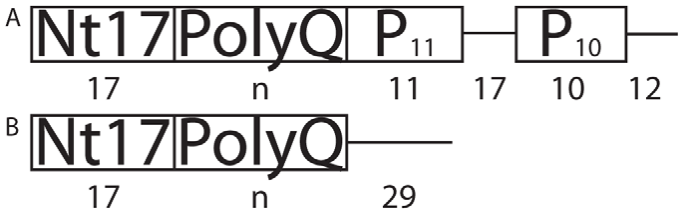
Constructs studied. Diagrams of the organization of the sequence regions in the (**a**) XN1 and (**b**) XN1-P_11_-P_10_ constructs are shown. The number of residues in each region is indicated below the region. For all three constructs (XN1, XN1-P_11_-P_10_ and the homopolymer Q_n_, not shown) we vary the number of repeats modeled: n = 23, 36, 40, 47. In (**a**) XN1, the first N-terminal 17 residues are collectively referred to as the Nt17 region. Following Nt17 is the polyQ region, which contains a variable *n* number of glutamine repeats. P_11_ and P_10_ are the regions of 11 and 10 proline repeats respectively; they are referred to as the polyP regions. A region of 17 residues tethers the polyP regions together. Finally, there are 12 residues in the C-terminus of XN1. The (**b**) XN1-P_11_-P_10_ construct is identical to XN1 with the exception that it does not contain the polyP regions. The XN1 sequence is explicitly written in Fig. 4d. We use the title of “construct” to refer to either XN1, XN1-P_11_-P_10_ or Q_n_. A “model” is a specific polypeptide, such as XN1Q_23_, which is a polypeptide of XN1 with 23 glutamine repeats. Thus a total of 12 models were studied, divided into 3 constructs with 4 different glutamine lengths.

Due to the structural complexity of XN1, previous experimental and computational studies have focused on specific regions of XN1 (Fig. 1). First, by studying polyQ homopolymers (Q_n_), and similar constructs, it has been shown that as the length increases, Q_n_ transitions from forming random coils in solution to β-sheets [16], [21]–[27]. Yet, at least one computational study finds no such transition [28]. Other authors find the formation of a β-helix [29]–[33], first proposed by Perutz [34]. Additionally, the polyQ region has been shown to unfold neighboring regions [20], [35]–[38], which suggests the flanking regions should be included to better model the behavior of XN1. Second, others [25], [39]–[41] have studied polyQ together with a polyproline domain, because XN1 has two polyproline (polyP) regions near the polyQ region (Fig. 1a). Experimental studies of these models, indicate that the polyP regions form polyproline type II (PPII) helices [3], [40], [41], which compete with the β-sheet structure of the polyQ region [40] and possibly protect against aggregation [1], [3], [5], [20], [39], [40], [42]–[44]. Last, recent models that include the first 17 residues (Nt17) of XN1 that are N terminal to the polyQ region have been studied [20], [42], [45]–[47]. By studying isolated Nt17 polypeptides, investigators have found that this region is either a compact random coil [20], an α-helix [42], [45] or both [20], [46], which resists aggregation [20], [46]. However, an Nt17+polyQ chimeric polypeptide was found to be highly prone to aggregation, more so than an unflanked Q_n_ homopolymer [20]. Thus, the structural role of the Nt17 region in XN1 aggregation is still unclear. Although the polyQ region has been individually shown to aggregate through studies on Q_n_, its effects are unknown in the context of the XN1 flanking regions. For example, it is uncertain whether the Nt17 region assists [3], [20], [45] or hinders [1], [46], [48] aggregation, and perhaps separately, to what extent the polyP region prevents aggregation. Therefore, it is necessary to study XN1 misfolding and aggregation in the context of flanking regions.

Despite the structural information about each region, there still lacks a complete picture of XN1 due to the non-additive effect of interactions between different structural elements. Here, we perform all-atom discrete molecular dynamics (DMD) [49]–[52] simulations to systematically study structural dynamics of XN1 and its variants. DMD has been shown to have a higher sampling efficiency than traditional molecular dynamics and has been used to study protein folding thermodynamics and protein aggregation [53]. All-atom DMD features a transferable force field and has been successfully used to fold several small proteins *ab initio* [52] and to study the folding and misfolding dynamics of Cu, Zn superoxide dismutase [54]. We construct 12 polypeptides (Fig. 1) categorized into three sets. In the first set, we study XN1 in its entirety (Fig. 1a) in order to capture the interactions between the Nt17, polyQ and polyP flanking regions simultaneously. In the second set, we study XN1 without the polyP flanking regions, titled XN1-P_11_-P_10_, (Fig. 1b) in order to determine the effect of the polyP regions on XN1 in the context of naturally occurring flanking regions. In the third set, we study polyQ homopolymers in the absence of all flanking regions (Q_n_) as controls. In each set, four different numbers of glutamine repeats are included: 23 (non-pathogenic), 36 (threshold), 40 and 47 (pathogenic). As originally suggested by Perutz [36] and expanded upon by others [1], [3], [20], [35], [36], [38], [55], the sequence context for a polyQ region can alter its aggregation mechanism; here, we find a more complete view of how the context plays a significant role in the secondary structure. In the context of XN1, the residues in the polyQ region have a lower probability of adopting β-sheet conformations, due to inhibition by the polyP regions. Surprisingly, by increasing the number of glutamine repeats in the polyQ region, the Nt17 region can be induced to fold into a β-strand. Thus, we suggest that the polyQ and flanking regions in XN1 are strongly coupled in XN1 folding and misfolding.

## Results

For each of the 12 models (Fig. 1), we perform replica exchange DMD simulations to efficiently sample the folding landscape of the polypeptides [56]–[60]. In each simulation, we start from a completely stretched conformation. We discard the first 0.5% of the trajectories, which have drastic energy and structural changes, to disregard the initial equilibration; we only use the equilibrated parts of the trajectories for analysis. First, we model XN1 (Fig. 1a) in its entirety to simultaneously study the interactions among all flanking regions, such as the Nt17, polyQ and polyP regions. Second, we model a mutant of XN1 that is missing the polyP regions: XN1-P_11_-P_10_ (Fig. 1b) to study the structural effects of the polyP regions, which have been shown to protect against XN1 aggregation. By comparing the results from the XN1 models to the XN1-P_11_-P_10_ models, we can determine the role of the polyP regions. Lastly, as controls, we model Q_n_ homopolymers in the absence of all flanking regions.

### XN1Q_n_ are less stable than Q_n_ and XN1Q_n_-P_11_-P_10_

**Figure 2:**
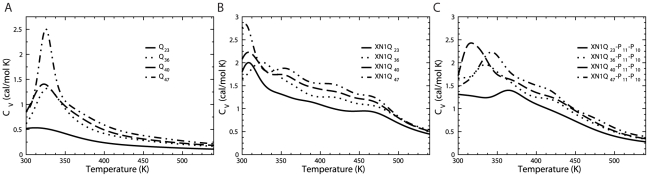
Thermodynamics of the peptides. Heat capacity and temperature curves are calculated with WHAM analysis of the simulation trajectories. (**a**) Calculations of the Q_23_, Q_36_, Q_40_ and Q_47_ models indicate the folding transition peaks become taller and narrower for longer glutamine lengths. (**b**) For the XN1Q_23_, XN1Q_36_ and XN1Q_47_ models, the transition temperatures are nearly identical (308K, 308K and 304K respectively). However, the XN1Q_40_ model has a larger transition temperature around 325K. (**c**) The XN1Q_23_-P_11_-P_10_, XN1Q_36_-P_11_-P_10_, XN1Q_40_-P_11_-P_10_ and XN1Q_47_-P_11_-P_10_ models have varied transition peaks. The transition temperatures are 365K, 335K, 317K and 343K respectively. Detailed data on the peak positions are found in Table S1.

We apply the weighted histogram analysis method (WHAM) to analyze the folding thermodynamics of all simulated peptide systems [61]. For each peptide model, we calculate the heat capacity (C_V_) at different temperatures (Fig. 2). We find Q_23_ undergoes a non-cooperative folding transition from an extended and unfolded state to a collapsed globule state, characterized by the broad and shallow C_V_ peak (Fig. 2a). Similar peaks, indicating a coiled-globule transition of a polyQ system, have been documented elsewhere [14]. As the length of Q_n_ increases, the heat capacity peak gets taller and narrower, suggesting an increased folding cooperativity. In all three constructs (XN1, XN1-P_11_-P_10_ and Q_n_) we find the transition temperature corresponding to the C_V_ peak (Fig. 2 and Table S1) is almost unaffected by the length of the polyQ region. Since the transition temperature is indicative of the polypeptide stability, our simulation suggests that the length of the polyQ region does not affect the stability of the polypeptides. Interestingly, we find that the average transition temperature for the XN1 models (311 K) is smaller than that of the Q_n_ (322 K) and the XN1-P_11_-P_10_ (340 K) models. The differences in transition temperatures indicate the XN1-P_11_-P_10_ models are the most stable, followed by the Q_n_ models and lastly, the XN1 models are the least stable.

### Q_n_ transitions from random coil to β-sheet

**Figure 3:**
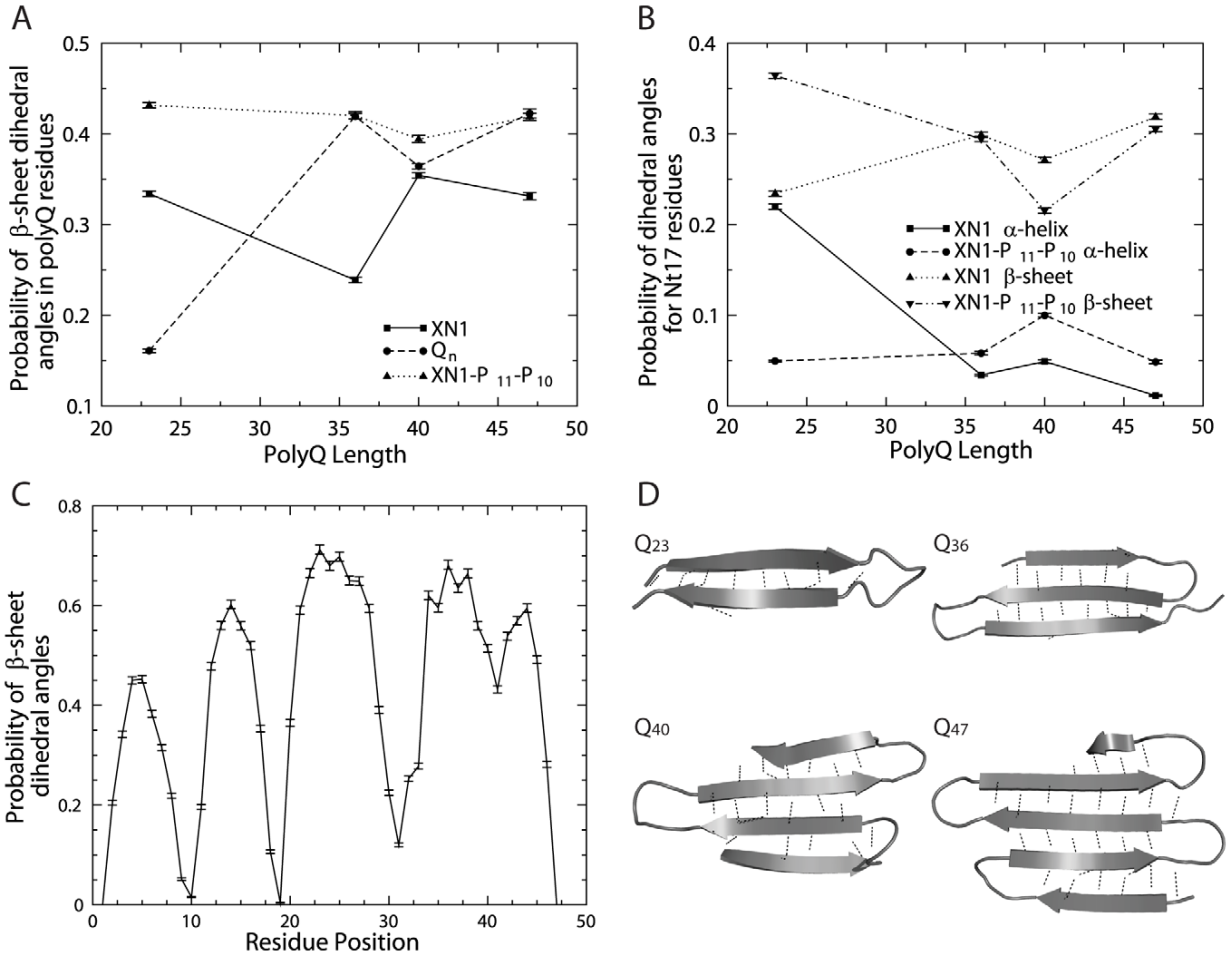
Secondary structure probabilities. Selected secondary structure probabilities of residues in the (**a**) polyQ region, the (**b**) Nt17 region and (**c**) each residue of Q_47_. Lastly, (**d**) representative structures of Q_n_. (**a**) In the XN1 and XN1-P_11_-P_10_ models, polyQ residues have an almost constant β-strand probability for varying number of glutamine repeats. For all lengths of polyQ in XN1, the polyQ residues have a 31%±4% β-strand probability. For all lengths of polyQ in XN1-P_11_-P_10_, the polyQ residues have a 42%±1% β-strand probability. In the context of Q_n_, however, the glutamine residues for long Q_n_ lengths have an increase in β-strand probability and a decrease in the random coil probability (data not shown). On average, for n = 36, 40 and 47, residues in the Q_n_ polypeptides are 9% more likely to adopt β-strand conformations than the polyQ residues in the XN1 polypeptides. For the same polyQ lengths, polyQ residues in XN1-P_11_-P_10_ and all the residues in Q_n_ have an average difference of less than 1% in β-strand probability. (**b**) We show the α-helix and β-strand probabilities of the Nt17 residues as the length of the neighboring polyQ region increases. For XN1Q_23_, we find the probabilities of forming an α-helix or β-strand are similar; the difference is less than 1%. However, when the polyQ length increases (XN1Q_36–47_), the difference becomes more than 20% in favor of a β-strand. Contrarily, we find that the Nt17 residues in XN1-P_11_-P_10_ models consistently prefer β-strand dihedral angles over α-helix dihedral angles; the difference is over 20% for each length of polyQ. (**c**) As an example, we present the probability of each residue in Q_47_ to have a β-sheet conformation. There are continuous stretches of high probabilities: residues 2–8, 11–17, 21–29 and 34–45. These stretches are likely the locations of continuous β-strands. The residues with surprisingly low β-sheet conformation are the location of turns between β-strands. The periodic shape of the graph indicates a β-sheet similar to the one in panel d). The average of all these probabilities is 42%; it is one data point in panel a). (**d**) These are representative structures of the Q_n_ polypeptides determined through clustering. Q_23_ shows a β-hairpin, and although this structure is from the largest cluster, it represents only 6% of all the structures used for clustering (see methods). Therefore, it is rare, but possible, for Q_23_ to adopt a β-hairpin conformation. The longer homopolymers are more likely to adopt a conformation similar to the ones depicted here; Q_36_, Q_40_ and Q_47_ represent 50%, 23% and 40% of their respective clustered structures (Table S3). Most strands are between 6 and 9 residues long. Intra-backbone hydrogen bonds are shown only for those residues forming β-strands. Secondary structures are automatically calculated by PyMOL and are not used to calculate probabilities.

As suggested by the Wetzel group and others [11], [13], [19], misfolding of polyQ monomers might initialize the aggregation and play a crucial role in the formation of amyloid fibrils. The peptide of polyQ is naturally unstructured, and thus, the length-dependent aggregation behavior can only be explained by the rare and spontaneous misfolding of the peptide. Therefore, we focus on the compact low-energy state from simulations (see Methods). The compact low-energy state usually constitutes only 13–27% of the total populations (Table S2). Among this subset of compact structures, we find a representative structure using a clustering algorithm (see Methods). These representative structures are not definitive misfolded states, but rather represent common, accessible compact states. We find that Q_n_ is able to form a β-sheet for all lengths modeled (Fig. 3d). As the length of Q_n_ increases, the β-sheet expands and contains more β-strands. Even the shortest polypeptide, Q_23_, is capable of forming a small β-sheet (a β-hairpin); although, this structure rarely forms during the simulation (Fig. 3d). Additionally, we calculate the probability of observing a given secondary structure for each residue (Fig. 3a,b,c) in the compact state (see Methods). For example, we find that in Q_47_ there are many segments of the polypeptide featuring high β-conformation (Fig. 3c). Although the probability is computed for each residue, a high probability of β-conformation for consecutive residues indicates the formation of a β-strand (Fig. 3d). Furthermore, an overall perspective is gained when this probability is averaged over multiple residues (Figs. 3a and 3b). We find the compact states of Q_23_ to be primarily unstructured; a typical residue in Q_23_ is a random coil 67% of the time. In contrast, the residues in the long Q_n_ models of Q_36–47_, have β-sheet dihedral angles 40% of the time. That is, residues in the long Q_n_ models adopt β-sheet conformations almost 2.5 times more often than Q_23_ (Fig. 3a). The relatively high β-sheet probabilities do not contradict the experimental observations of little to no monomeric β-sheet structure [19], [15], [25], since the secondary structure probability calculation is computed only for the small subset of compact and low-energy states. Additionally, the structural ensembles are constructed from the replica exchange simulations, which cover a wide-range of temperatures (see Methods). As a result, the distribution of conformations does not correspond to a single experimental condition, and thus, cannot be compared with experimental measurements. However, these ensembles can be used to evaluate the structural propensities of various polyQ constructs. From our calculations of the compact polypeptide, we find that Q_n_ residues tend to transition from random coil conformations at short lengths, to β-sheet conformations at long lengths.

### PolyP hinders β-sheet formation of polyQ in XN1

**Figure 4:**
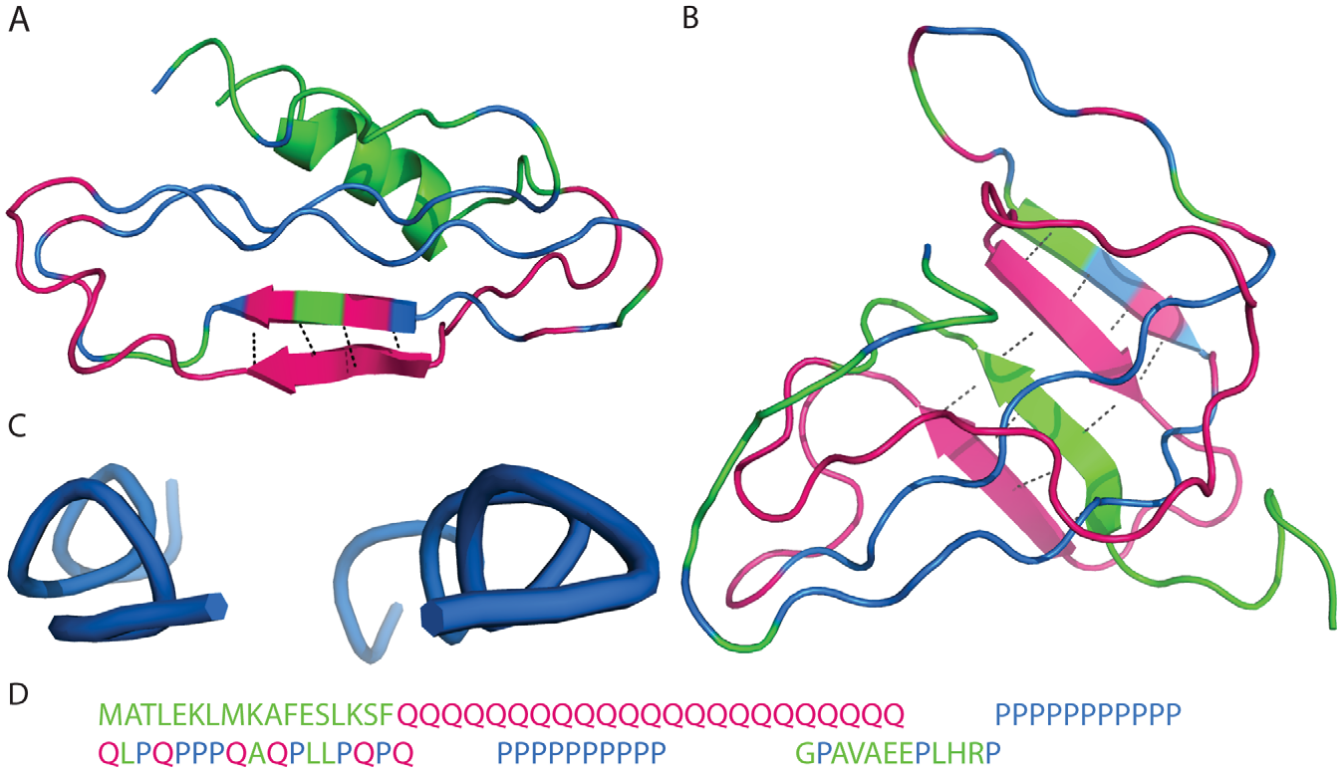
Representative structures of XN1. Structures that are representative of (**a**) XN1Q_23_ and (**b**) XN1Q_47_ are shown. A detailed view of the polyP regions from the XN1Q_23_ structure is also presented (**c**). Pink residues are glutamines; blue residues are prolines, and all other residues are colored green. The sequence for XN1 is shown (**d**) for n = 23 as in panel **a**). The coloring in the sequence matches the coloring of the polypeptide backbones in both structures. The (**a**) XN1Q_23_ and (**b**) XN1Q_47_ structures respectively represent 37% of 1036 clustered structures and 17% of 1032 clustered structures (see methods and Table S3). From these example conformations, we see some structural correlations that complement the secondary structure probability calculations (Fig. 3). First, no drastic change in the β-strand structure of the polyQ region is seen between the XN1Q_23_ and XN1Q_47_ structures. Second, the α-helix in the Nt17 region of (**a**) XN1Q_23_ has transformed into a β-strand in (**b**) XN1Q_47_. Third, the two polyP regions form (**c**) PPII helices. The probability data (not shown) indicates that these PPII helices exist in 100% of the partially folded structures. The intra-main chain hydrogen bonds are shown for those residues forming β-strands. As in Fig. 3d, the secondary structures are assigned by PyMOL. (**d**) The sequence is divided into the same regions as outlined in Fig. 1a.

In the XN1 model, the polyQ region is surrounded by multiple flanking regions (Fig. 1), including the Nt17 and polyP regions. We find that for n = 36, 40 and 47, the residues in the polyQ region adopt β-sheet conformations approximately 10% less frequently than residues in isolated Q_n_ (Fig. 3a). Thus, the flanking regions can lower the probability of the residues in the polyQ region to adopt β-sheet conformations. Based on secondary structure probabilities (data not shown) and the corresponding representative structures (Figs. 4a, 4b), we find that the two polyP stretches in XN1 consistently form PPII helices (Fig. 4c). It is unlikely that the polyP regions fold into PPII helices due to interactions from neighboring regions. This is because the PPII helices are consistently found in every one of the compact structures; whereas, the other regions have more variable secondary structures. Thus, the polyP regions are likely forming PPII helices independently. We hypothesize that the P_11_ and P_10_ regions dominate the fold of XN1, by forming these PPII helices, and subsequently, the remaining regions, including the polyQ region, are affected by these two PPII helices.

In order to study the effect of the PPII helices on the polyQ region, we perform simulations of XN1-P_11_-P_10_ polypeptides that are sequentially identical to XN1 but lack the polyP regions (Fig. 1b). We find that residues in the polyQ region of XN1-P_11_-P_10_ models adopt β-sheet conformations 10% more often than similar polyQ residues in the XN1 models (Fig. 3a). In fact, for n = 36, 40 and 47, the polyQ residues in the XN1-P_11_-P_10_ and Q_n_ models have nearly equal β-sheet probabilities (Fig. 3a). Thus, the decrease in the probability of the polyQ region to form β-sheets in XN1 may be attributed to the polyP regions that form PPII helices. This relationship is consistent with experimental observations which show the polyP regions inhibit polyQ aggregation and toxicity [1], [3], [5], [20], [39], [40], [42]–[44].

### Nt17 can be induced into a β-strand

It has been experimentally shown that the Nt17 region can be a tight random coil [20], [46] or an α-helix [20], [45], [46], [48]. However, once fused to the polyQ region, the Nt17 region promotes the aggregation of polyQ [20], [47]. To investigate the impact of the Nt17 region in XN1 misfolding, we study the Nt17 region in the context of XN1. From secondary structure probabilities of the XN1 and XN1-P_11_-P_10_ models, we find the Nt17 residues adopt random coil conformations over 50% of the time, regardless of the polyQ length. This observation is consistent with the previous [20] study in that the Nt17 region is most likely to be a random coil in the context of XN1. We also characterize the α-helix and β-sheet conformation probabilities for the Nt17 residues. For the XN1 models, we find that the likelihood of Nt17 residues forming an α-helix or β-strand conformation is correlated with the length of the neighboring polyQ region (Fig. 3b). In the shortest model, XN1Q_23_, the residues in the Nt17 region are equally likely to adopt an α-helix or β-sheet conformation. However for longer models, XN1Q_36–47_, the Nt17 residues adopt a β-sheet conformation at least six times more often than an α-helix conformation. In fact, for the longest model, XN1Q_47_, the Nt17 residues sample β-sheet dihedral angles 30 times more frequently than α-helix dihedral angles (Fig. 3b). The representative structures of the XN1Q_23_ and XN1Q_47_ models depict an example of this transition from an α-helix to a β-strand (Figs. 4a and 4b). Thus, in the context of XN1, we find that as the polyQ length increases, the Nt17 residues significantly prefer a β-sheet conformation over an α-helix conformation. That is, by elongating the polyQ region, the Nt17 region can be induced into a β-strand.

In the absence of the polyP regions, the Nt17 residues have different secondary structure probabilities. First, for all lengths of polyQ, the Nt17 residues in the XN1-P_11_-P_10_ models show a preference for adopting β-sheet rather than α-helix conformations. Even for the shortest polyQ length, the Nt17 residues prefer β-sheet dihedral angles (Fig. 3b). Thus, at least in the case of 23 glutamine repeats, the Nt17 region must be either directly or indirectly affected by the polyP regions; such that, by removing the polyP regions, the Nt17 region is more likely to form a β-strand. Second, for n = 36, 40 and 47, the Nt17 residues are nearly equally likely to adopt β-sheet conformations in either the XN1 or XN1-P_11_-P_10_ constructs (Fig. 3b). Therefore, for long polyQ lengths, the polyP regions in XN1 have little effect on the β-sheet probability of the Nt17 region.

## Discussion

### The length dependence of Q_n_ aggregation

One intriguing phenomena of glutamine expansion diseases is the length dependence of disease onset [2], [9]. It has been suggested that both short and long Q_n_ polypeptides can access similar misfolded structures that lead to aggregation, but the frequency at which this misfolded structure is visited depends on the length of Q_n_ [62]. That is, long Q_n_ aggregate fast, because they misfold frequently; contrarily, short Q_n_ rarely misfold and thus aggregate slow. Mounting experimental evidences support this model by showing that, regardless of length, Q_n_ polypeptides form aggregates of similar structure [25], [27], [28], [39], [62], which suggests the misfolded structures for all Q_n_ are also similar. Additionally, kinetic studies [16], [21]–[27] verify the correlation between the length of the Q_n_ polypeptide and the rate at which it aggregates in solution. The remaining question is to determine the common misfolded structure that leads to aggregation. To this end, some investigators [8], [11], [16]–[18] have suggested that early forms of Q_n_ aggregates have high amounts of β-sheets. In the previous investigation by Wetzel's group, [11] glutamine homopolymers were capped by flanking lysine residues (K_2_Q_n_K_2_) to increase the peptide solubility [63]. It has been argued that the electrostatic repulsion between flanking lysines might prevent the formation of compact structures and alter the aggregation kinetics of the peptide system [19]. As the length increases, the screening effect reduces. Hence, despite the screening effect, the observation of intrinsic β-sheets formation of polyQ peptides is still valid. Our resulting extended β-sheet structures are prone to aggregation with exposed hydrogen bond donors and acceptors found in the polypeptide backbone [64]. By seeking to satisfy these bonds, the polypeptides can form bonds with other polypeptides that similarly have an exposed backbone, leading to the formation of large aggregates.

Our simulations of Q_n_ support a model, similar to one outlined by [27], wherein a common, compact misfolded state is accessible to most Q_n_ monomers and rates of misfolding are length-dependent. Accordingly, we find that partially folded monomers of both short and long Q_n_ polypeptides can have high amounts of β-character (Fig. 3d). Additionally, we find that the residues in long Q_n_ models, with 36 or more repeats, are more likely to have β-sheet conformations than short models, Q_23_ (Fig. 3a). The secondary structure probability of each individual residue is proportional to the probability of the overall polypeptide adopting that secondary structure. Thus we find a positive correlation between the length of the Q_n_ polypeptide and its probability of forming a β-sheet. Because previous studies [8], [11], [16]–[18] have linked β-sheet formation to aggregation, we suggest that long Q_n_ polypeptides are therefore more likely to form aggregates.

### The polyP regions protect XN1 from aggregation

Recent studies [1], [3], [5], [20], [39], [40], [42]–[44] indicate that the addition of a polyP region can inhibit aggregation of the polyQ region; this inhibition has been associated [40] with a PPII helix structure in the polyP region. We are able to find a structural effect on the polyQ region from the formation of PPII helices in the polyP regions. We find that the polyP regions form PPII helices and suppress the probability of polyQ residues in XN1 to adopt β-sheet dihedral angles. This suppression is still present for long polyQ lengths that are associated with disease. Upon removing these PPII helices, we find the polyQ residues are more likely to adopt β-sheet conformations; the likelihood is similar to that of the isolated glutamine homopolymers: Q_n_ (Fig. 3a). This similarity indicates that the other flanking regions of XN1 have little effect on the probability of the polyQ region to form a β-strand. Furthermore, by considering that β-sheet formation has been linked to aggregation, we find that our results reflect experimental results. That is, because the polyP regions decrease the probability of polyQ residues adopting β-strand conformations, these polypeptides have a slower rate of aggregation, which is seen in other experiments [1], [3], [5], [20], [39], [40], [42]–[44]. Additionally, the polyP regions greatly destabilize XN1 polypeptides (Fig. 2b and 2c). Since unfolded polypeptides in the random coil state are unlikely to organize as an aggregate, the presence of the polyP in the XN1 sequence prevent it from folding into the aggregation-prone state. Therefore, we hypothesize that polyP regions protect XN1 from aggregation in two ways: 1) destabilize the polypeptide, and 2) the PPII helices formed by polyP inhibit formation of β-sheets.

### Nt17 misfolding in monomeric XN1

Currently, the role of the flanking regions in XN1 and other polyglutamine diseases is under debate [1], [3], [20], [45]–[48]. One model [3], [47] suggests that aggregation is initiated by the flanking Nt17 region. That is, initially, these flanking regions misfold and form oligomers; subsequently, there is an increase in the local concentration of the polyQ region, which causes the polyQ regions to misfold into protofibrils and ultimately mature, fatal fibrils. Others, however, contend that the native structure of the flanking regions is one that resists aggregation [1], [46], [48]. In a recent study [20], the expansion of polyQ repeats in XN1 is found to promote the misfolding of Nt17, which leads to rapid formation of oligomers. In particular, the structure of the Nt17 flanking region in XN1 is one part of this debate, which we discuss here.

We find a sharp decrease in the probability of the Nt17 residues to adopt α-helix conformations as the length of the polyQ region increased. Concurrently, however, these residues are more likely to have β-strand conformations for longer polyQ repeat lengths (Fig. 3b). Hence, our results indicate misfolding of the Nt17 region; that is, the α-helical native structure misfolds into a β-strand in the pathogenic associated XN1 models. Our simulation of the XN1Q_47_ suggests that both the Nt17 and polyQ regions simultaneously form β-strands (Fig. 4b). A possible future direction would be to identify the temperature at which the Nt17 residues transition from α-helical to β-sheet conformation; a similar calculation has been done elsewhere [65]. In terms of the problem of the aggregation mechanism of XN1, the next step is to determine the role these two regions play in oligomerization of XN1. Here, our computational study suggests that the polyQ region also plays a critical role in the early stages of aggregation [20].

## Methods

### Discrete molecular dynamics

Unlike traditional molecular dynamic simulations, we discretize the spherically symmetric, pair-wise interaction potential in a DMD simulation [49]–[52], where, the continuous potential between any two atoms is reduced to a series of square well potentials. Such a simplification considerably accelerates the computation time because in square well potentials, the particles do not experience any force except at the boundaries of the square wells. Thus, the particles travel with constant momenta until a boundary is reached; at such a boundary, the two particles experience a force and are considered colliding. At a collision, the momenta, angular momenta and energies of only the two colliding particles are updated according to conservation laws. Thus, the most computationally intense process is to sort the event list to determine the next collision. Further details on the particular version of DMD used here, such as interaction strength between atoms, are provided in reference [52]. Each polypeptide begins in an extended conformation; to allow for equilibration, the first 500 time units wherein the polypeptide has drastic energy and structural changes are discarded. We simulate each polypeptide in a cube with periodic boundaries. The dimension of the box is chosen to be large enough to fit the extended polypeptide.

### All-atom models of Q_n_, XN1 and XN1-P_11_-P_10_

We model each polypeptide using an united all-atom approach, which is explained in detail elsewhere [52]. Briefly, this approach models all heavy atoms and polar hydrogen atoms in a polypeptide; interactions between atoms are governed by the Medusa Force Field [66]. The interactions include van der Waals (VDW) based on CHARMM19, orientation-dependent hydrogen bonding and implicit solvation EEF1 [67]. Because electrostatic interactions at long distances are weakened due to solvent screening, we currently do not model these effects in the all-atom DMD. Salt bridges between side chains are captured partially through the hydrogen bonding potential [52]. Despite the approximation of electrostatic interactions, we were able to fold six small proteins to their native state *ab initio* [52]. The sequence used to model XN1 is taken as the first 90 residues in Human Huntingtin Protein from NCBI [68] (Fig. 4d). There are charged, polar and non-polar groups scattered throughout the sequence. Additionally, the particular sequence, taken from NCBI, contains 23 glutamine repeats, which is a non-pathogenic length. The sequence is not modified except to add glutamines in the polyQ region as indicated or to remove the P_11_ and P_10_ stretches in the XN1-P_11_-P_10_ models.

### Replica exchange DMD simulations

To efficiently sample protein conformations, we use the replica exchange simulation technique [56]–[60]. With this technique, we are able to utilize multiple, parallel simulations of identical systems called replicas. For each of the 12 polypeptides, we perform simulations on eight replicas with the following set of temperatures: {0.85, 0.75, 0.68, 0.64, 0.6, 0.57, 0.53, 0.5}. The temperature units are in kcal/mol/k_B_, or about 500K. At a regular time interval of 500 time units (approximately 25 ps), we consider exchanging the temperature of two replicas. We only allow an exchange for two replicas with neighboring temperatures, for example 0.6 and 0.64. We use a Monte-Carlo based approach to accept or reject an exchange. The simulation length of each replica is 1×10^6^ time units (∼50 ns).

### Structure screening

Replica exchange simulations allow us to efficiently sample the conformational space of XN1 and its variants by simulating a wide range of temperatures. However, we focus only on the compact structures of the polypeptides. To screen for these compact states, we eliminate highly extended structures, which are those with a large radius of gyration (R_g_), and we include only those with low energy. The former criterion eliminates the transient structures explored during the early stages of folding when the polypeptide is far from its favored structure. The latter criterion selects for structures further along the folding pathway, because polypeptides lose energy during folding. We determine both the R_g_ cutoff (Fig. S1) and energy cutoff (Fig. S2) from a histogram of conformations sampled during the simulation. Each simulation produces 800,000 structures (1 conformation per 10 time units per replica). From this entire set, a subset of roughly 100,000–200,000 structures is selected through this screening process (Table S2). The energy cutoff is chosen to select for the lowest energy Gaussians.

### Secondary structure probabilities

The probabilities calculated here are averages over the compact ensemble, which is a small subset of the entire population (Table S2). Thus, the calculations do not describe the polypeptides in general. Instead, the secondary structure likelihoods describe the polypeptides in a partially folded state, which estimates the misfolded structure. For each of the compact structures, we can calculate the backbone dihedral angles (ϕ & ψ) and the corresponding secondary structure for each residue. Then from the ensemble of compact structures for each polypeptide model, we compute the probability of a given residue to adopt α-helix, β-sheet, turn or random coil dihedral angles (Fig. 3c). Furthermore, we also determine the secondary structure probability of a set of residues, or a region, by averaging the secondary structure probability over those residues (Figs. 3a, 3b). For example, a β-strand probability of 0.3 in the polyQ region means that on average, a given residue in the polyQ region has a 30% chance of adopting β-strand dihedral angles. These probabilities are calculated for individual amino acids regardless of neighboring residues. However, consecutive residues with high amount of calculated secondary structure probability will suggest the probability of forming specific secondary structures. Thus, this analysis method allows for comparison of secondary structure tendencies for polypeptides based on the compact ensemble. To calculate the error, we compute the standard error for each value; where the number of events is the number of times the energy of the trajectory crossed the energy cutoff (Fig. S3). In effect, this method counts the number of times during the folding trajectory that the polypeptide enters or exits the compact domain of its folding landscape.

### Clustering

For visualization purposes, we identify representative structures for each polypeptide using an hierarchical clustering algorithm [69]. The structures used for clustering are taken from the simulation trajectories and are separated by at least 50ps. For most of the models studied, roughly 1500–2000 structures are used for clustering. As exceptions, the XN1Q_23_ and Q_23_ models include only about 1030 structures. The number of clusters is different for each polypeptide model and varied from 101–843. Similarly, the population of the largest cluster also varied. A detailed summary of these values for each polypeptide model is presented in the supplemental materials (Table S3). In this clustering scheme, the nodes are structures and the distance between a pair of nodes is the root mean square distance (RMSD) between the two structures. We use the single-linkage or minimum distance criterion for clustering. That is, the distance between a node and a cluster is equal to the distance between that node and the closest node in the cluster. The cutoff distance determines the size of clusters. If the distance between a node and a cluster is less than the cutoff, we include the node in that cluster; otherwise, we exclude the node from that cluster. We use a RMSD cutoff of 2.5Å for clustering the XN1Q_47_-P_11_-P_10_ polypeptide and a 2Å RMSD cutoff for all other polypeptides. These cutoffs are chosen to maintain high structural similarity among the nodes in a cluster (Fig. S4). We study the centroid of each cluster, which is the most representative node or the centermost node of a cluster. Furthermore, we select the centroids from the largest clusters to be overall representatives of their respective polypeptides. To gauge the significance or reliability of the centroid, we calculate the ratio of the number of structures present in its cluster and the total number of structures considered for clustering. Example calculations are reported in the captions of Figs. 3d and 4. Finally, necessary raw data is presented in Table S3.

## Supporting Information

Table S1 Thermodynamic Peak Values. Data corresponding to the peaks (Fig. 2) of the heat capacity versus temperature curves for each polypeptide modeled. Column 2 contains the values from the position of the peaks, which is the temperature at which the transition from folded to unfolded occurs. Column 3 contains the corresponding heat capacities at the folding transitions. Increasing glutamine repeat length does not correlate significantly with the transition temperature. The XN1 and Qn models show higher heat capacities for longer glutamine repeats.
(0.03 MB DOC)

Table S2 Compact Structure Populations. Some statistics on the populations of compact structures that are used for analysis. Each simulation produces 800,000 structures in total. A subset of compact, low-energy structures is selected for analysis. Here, the total number of these compact structures is given for each simulation. For comparison, the percentages out of the total 800,000 structures represented by the compact populations are also shown. Because the compact populations are only 30% or less of the total, the polypeptides are primarily extended and unstructured.
(0.03 MB DOC)

Table S3 Clustering Data. Statistics describing the clustering results. For each polypeptide studied, the following values are shown: total number of structures used for clustering (column 2), number of clusters (column 3) and the population of the largest cluster (column 4). A Root Mean Square Deviation (RMSD) cutoff of 2Å is used for all polypeptides, except XN1Q47-P11-P10 where 2.5Å is used. Due to computational constraints, only about 1% of all compact structures (Table S2) are used for clustering. The structures presented in Figures 3d and 4 are centroids of the largest cluster.
(0.03 MB DOC)

Figure S1 Example Rg Histogram. Distribution of the Radius of Gyration (Rg). This histogram is an example from one polypeptide (XN1Q23). The bin size is 1Å. Exactly 800,000 Rg values, one per 10 time units per replica, from the trajectories of all eight replicas are used to generate the distribution. The dashed line indicates the Rg cutoff value (20Å in this case), which is used to determine compact structures. Structures with a larger Rg than the cutoff, are considered extended or insufficiently compact and are not included in the compact ensemble used for further analysis. Note the sharp peak around 16Å; most polypeptides produced a similar sharp peak. This peak indicates that most conformations explored during the simulation are compact.
(0.07 MB DOC)

Figure S2 Example Energy Histogram. Example histogram of two replicas from one polypeptide (XN1Q23). The bin size is 1 cal/mol. The distribution for each replica is based on 1,000,000 data points: one value per time unit. Here, the data from two replicas are pooled; therefore this distribution is based on 2,000,000 data points. The dashed line indicates the energy cutoff value (−285 cal/mol); this cutoff applies to all replicas of this polypeptide. The cutoff is chosen such that only the lowest energy states are included as compact structures.
(0.08 MB DOC)

Figure S3 Trajectories Sampling the Compact Domain. Two example trajectories that depict how the simulations explored states that are compact and partially folded. Both trajectories are from the XN1Q23 simulation, and each trajectory corresponds to the particular replica indicated in the legend. The energy cutoff value is indicated as the red line. This value is determined by a histogram (Fig. S2). All explored states that occur below the cutoff are considered compact and are included in the compact ensemble.
(0.13 MB DOC)

Figure S4 Hierarchical Clustering. One example (from XN1Q23) of how the cutoff distance for clustering is determined. With hierarchical clustering, we are able to determine the number of clusters remaining for a given Root Mean Square Deviation (RMSD) cutoff. The cutoff value, indicated by the dashed line (2Å), is chosen to maximize the clustering of similar structures while avoiding clustering of unlike structures. Clustering of similar structures occurs in the steeply descending portion of the curve (between 1Å and 2Å). In this region clusters are close to each other or similar, because a very small increase in the cutoff distance joins two clusters. Clustering of structures that are less similar occurs in the tail end of the curve (roughly 3Å or longer). Here, the clusters are distant or dissimilar and a large increase in the cutoff distance is required to join two clusters. Thus, a suitable cutoff is often found after the most similar clusters have been joined (here, between 1Å–1.5Å) and before the distant clusters are joined (2.5Å and longer).
(0.08 MB DOC)
