# Supplementary material for: Polyglutamine Induced Misfolding of Huntingtin Exon1 is Modulated by the Flanking Sequences
Source: PLoS Comput Biol. 2010 Apr 29;6(4):e1000772. doi: 10.1371/journal.pcbi.1000772 (PMC2861695; doi:10.1371/journal.pcbi.1000772)
Supplement: Table S1 — Thermodynamic Peak Values. Data corresponding to the peaks (Fig. 2) of the heat capacity versus temperature curves for each polypeptide modeled. Column 2 contains the values from the position of the peaks, which is the temperature at which the transition from folded to unfolded occurs. Column 3 contains the corresponding heat capacities at the folding transitions. Increasing glutamine repeat length does not correlate significantly with the transition temperature. The XN1 and Qn models show higher heat capacities for longer glutamine repeats. (0.03 MB DOC) [file pcbi.1000772.s001.doc]

**Table S1. Thermodynamic Peak Values.** Data corresponding to the peaks (Fig. 2) of the heat capacity versus temperature curves for each polypeptide modeled. Column 2 contains the values from the position of the peaks, which is the temperature at which the transition from folded to unfolded occurs. Column 3 contains the corresponding heat capacities at the folding transitions. Increasing glutamine repeat length does not correlate significantly with the transition temperature. The XN1 and Q_n_ models show higher heat capacities for longer glutamine repeats.

| Polypeptide | T_transition_ (K) | C_V_^max^ (cal/mol K) |
| --- | --- | --- |
| XN1Q_23_ | 308 | 2.00 |
| XN1Q_36_ | 325 | 2.01 |
| XN1Q_40_ | 308 | 2.23 |
| XN1Q_47_ | 304 | 2.84 |
| Q_23_ | 312 | 0.53 |
| Q_36_ | 328 | 1.32 |
| Q_40_ | 323 | 1.41 |
| Q_47_ | 325 | 2.51 |
| XN1Q_23_-P_11_-P_10_ | 365 | 1.40 |
| XN1Q_36_-P_11_-P_10_ | 335 | 1.96 |
| XN1Q_40_-P_11_-P_10_ | 317 | 2.43 |
| XN1Q_47_-P_11_-P_10_ | 343 | 2.23 |
