# Supplementary material for: Polyglutamine Induced Misfolding of Huntingtin Exon1 is Modulated by the Flanking Sequences
Source: PLoS Comput Biol. 2010 Apr 29;6(4):e1000772. doi: 10.1371/journal.pcbi.1000772 (PMC2861695; doi:10.1371/journal.pcbi.1000772)
Supplement: Table S2 — Compact Structure Populations. Some statistics on the populations of compact structures that are used for analysis. Each simulation produces 800,000 structures in total. A subset of compact, low-energy structures is selected for analysis. Here, the total number of these compact structures is given for each simulation. For comparison, the percentages out of the total 800,000 structures represented by the compact populations are also shown. Because the compact populations are only 30% or less of the total, the polypeptides are primarily extended and unstructured. (0.03 MB DOC) [file pcbi.1000772.s002.doc]

**Table S2. Compact Structure Populations.** Some statistics on the populations of compact structures that are used for analysis. Each simulation produces 800,000 structures in total. A subset of compact, low-energy structures is selected for analysis. Here, the total number of these compact structures is given for each simulation. For comparison, the percentages out of the total 800,000 structures represented by the compact populations are also shown. Because the compact populations are only 30% or less of the total, the polypeptides are primarily extended and unstructured.

| Polypeptide | Compact structures | Percent from simulation |
| --- | --- | --- |
| XN1Q_23_ | 104567 | 13.1 |
| XN1Q_36_ | 191694 | 24.0 |
| XN1Q_40_ | 172590 | 21.6 |
| XN1Q_47_ | 105159 | 13.1 |
| Q_23_ | 176929 | 22.1 |
| Q_36_ | 196993 | 24.6 |
| Q_40_ | 156631 | 19.6 |
| Q_47_ | 180577 | 22.6 |
| XN1Q_23_-P_11_-P_10_ | 201177 | 25.1 |
| XN1Q_36_-P_11_-P_10_ | 190101 | 23.8 |
| XN1Q_40_-P_11_-P_10_ | 203819 | 25.5 |
| XN1Q_47_-P_11_-P_10_ | 220769 | 27.6 |
