# Supplementary material for: Polyglutamine Induced Misfolding of Huntingtin Exon1 is Modulated by the Flanking Sequences
Source: PLoS Comput Biol. 2010 Apr 29;6(4):e1000772. doi: 10.1371/journal.pcbi.1000772 (PMC2861695; doi:10.1371/journal.pcbi.1000772)
Supplement: Table S3 — Clustering Data. Statistics describing the clustering results. For each polypeptide studied, the following values are shown: total number of structures used for clustering (column 2), number of clusters (column 3) and the population of the largest cluster (column 4). A Root Mean Square Deviation (RMSD) cutoff of 2Å is used for all polypeptides, except XN1Q47-P11-P10 where 2.5Å is used. Due to computational constraints, only about 1% of all compact structures (Table S2) are used for clustering. The structures presented in Figures 3d and 4 are centroids of the largest cluster. (0.03 MB DOC) [file pcbi.1000772.s003.doc]

**Table S3. Clustering Data.** Statistics describing the clustering results. For each polypeptide studied, the following values are shown: total number of structures used for clustering (column 2), number of clusters (column 3) and the population of the largest cluster (column 4). A Root Mean Square Deviation (RMSD) cutoff of 2Å is used for all polypeptides, except XN1Q_47_-P_11_-P_10_ where 2.5Å is used. Due to computational constraints, only about 1% of all compact structures (Table S2) are used for clustering. The structures presented in Figures 3d and 4 are centroids of the largest cluster.

| Polypeptide | Structures | Clusters | Largest Cluster pop. |
| --- | --- | --- | --- |
| XN1Q_23_ | 1036 | 101 | 387 |
| XN1Q_36_ | 1894 | 411 | 179 |
| XN1Q_40_ | 1707 | 384 | 342 |
| XN1Q_47_ | 1032 | 163 | 173 |
| Q_23_ | 1757 | 843 | 113 |
| Q_36_ | 1959 | 291 | 981 |
| Q_40_ | 1554 | 241 | 359 |
| Q_47_ | 1795 | 155 | 727 |
| XN1Q_23_-P_11_-P_10_ | 2001 | 613 | 278 |
| XN1Q_36_-P_11_-P_10_ | 1890 | 514 | 272 |
| XN1Q_40_-P_11_-P_10_ | 2016 | 479 | 846 |
| XN1Q_47_-P_11_-P_10_ | 2187 | 505 | 153 |
