# Supplementary material for: Polyglutamine Induced Misfolding of Huntingtin Exon1 is Modulated by the Flanking Sequences
Source: PLoS Comput Biol. 2010 Apr 29;6(4):e1000772. doi: 10.1371/journal.pcbi.1000772 (PMC2861695; doi:10.1371/journal.pcbi.1000772)

**Figure S1. Example R_g_ Histogram.** Distribution of the Radius of Gyration (Rg).  This histogram is an example from one polypeptide (XN1Q_23_).  The bin size is 1Å. Exactly 800,000 R_g_ values, one per 10 time units per replica, from the trajectories of all eight replicas are used to generate the distribution.  The dashed line indicates the R_g_ cutoff value (20Å in this case), which is used to determine compact structures. Structures with a larger R_g_ than the cutoff, are considered extended or insufficiently compact and are not included in the compact ensemble used for further analysis. Note the sharp peak around 16Å; most polypeptides produced a similar sharp peak. This peak indicates that most conformations explored during the simulation are compact.


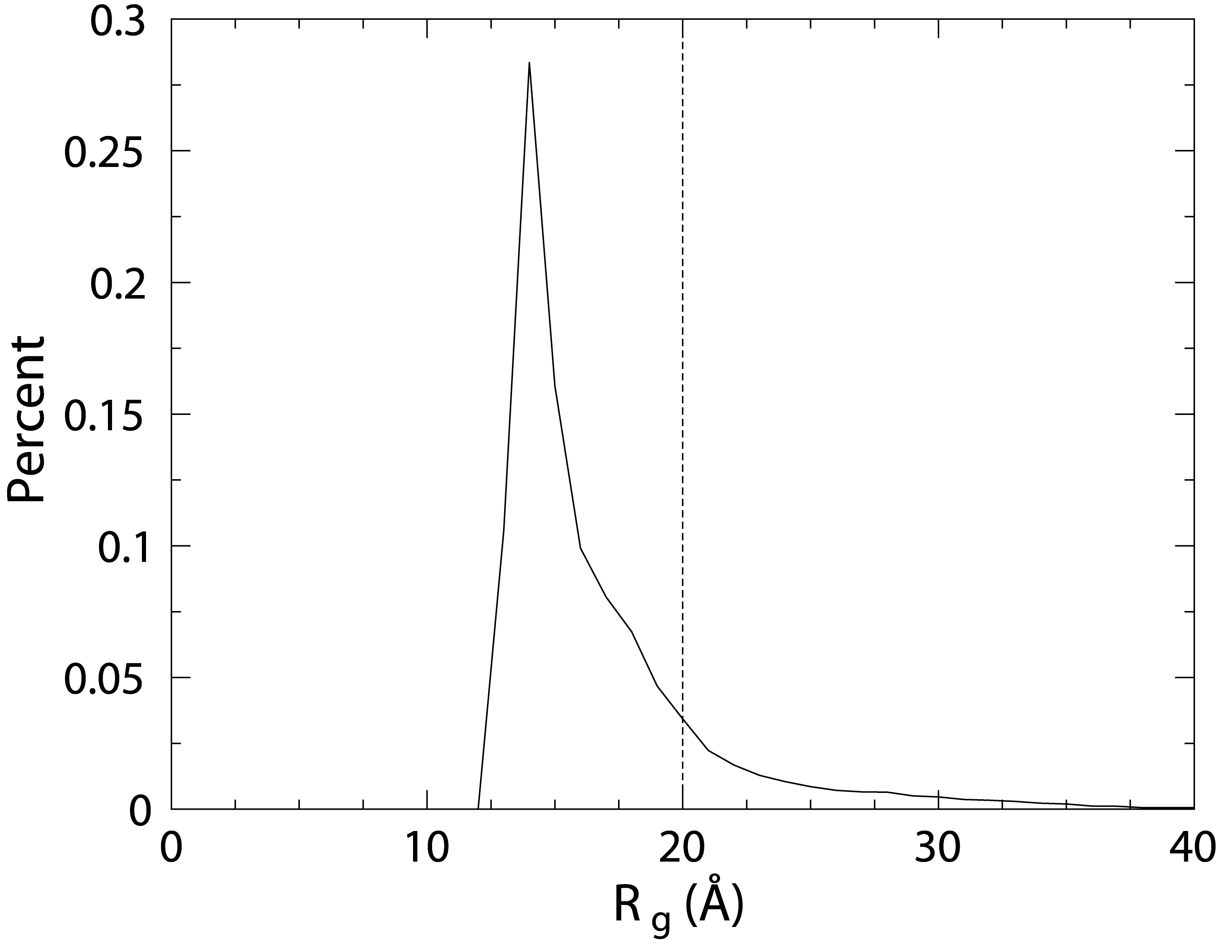

Supplement: Figure S1 — Example Rg Histogram. Distribution of the Radius of Gyration (Rg). This histogram is an example from one polypeptide (XN1Q23). The bin size is 1Å. Exactly 800,000 Rg values, one per 10 time units per replica, from the trajectories of all eight replicas are used to generate the distribution. The dashed line indicates the Rg cutoff value (20Å in this case), which is used to determine compact structures. Structures with a larger Rg than the cutoff, are considered extended or insufficiently compact and are not included in the compact ensemble used for further analysis. Note the sharp peak around 16Å; most polypeptides produced a similar sharp peak. This peak indicates that most conformations explored during the simulation are compact. (0.07 MB DOC) [file pcbi.1000772.s004.doc]
