# Supplementary material for: Polyglutamine Induced Misfolding of Huntingtin Exon1 is Modulated by the Flanking Sequences
Source: PLoS Comput Biol. 2010 Apr 29;6(4):e1000772. doi: 10.1371/journal.pcbi.1000772 (PMC2861695; doi:10.1371/journal.pcbi.1000772)

**Figure S2. Example Energy Histogram.** Example histogram of two replicas from one polypeptide (XN1Q_23_).  The bin size is 1 cal/mol.  The distribution for each replica is based on 1,000,000 data points: one value per time unit. Here, the data from two replicas are pooled; therefore this distribution is based on 2,000,000 data points. The dashed line indicates the energy cutoff value (-285 cal/mol); this cutoff applies to all replicas of this polypeptide. The cutoff is chosen such that only the lowest energy states are included as compact structures.


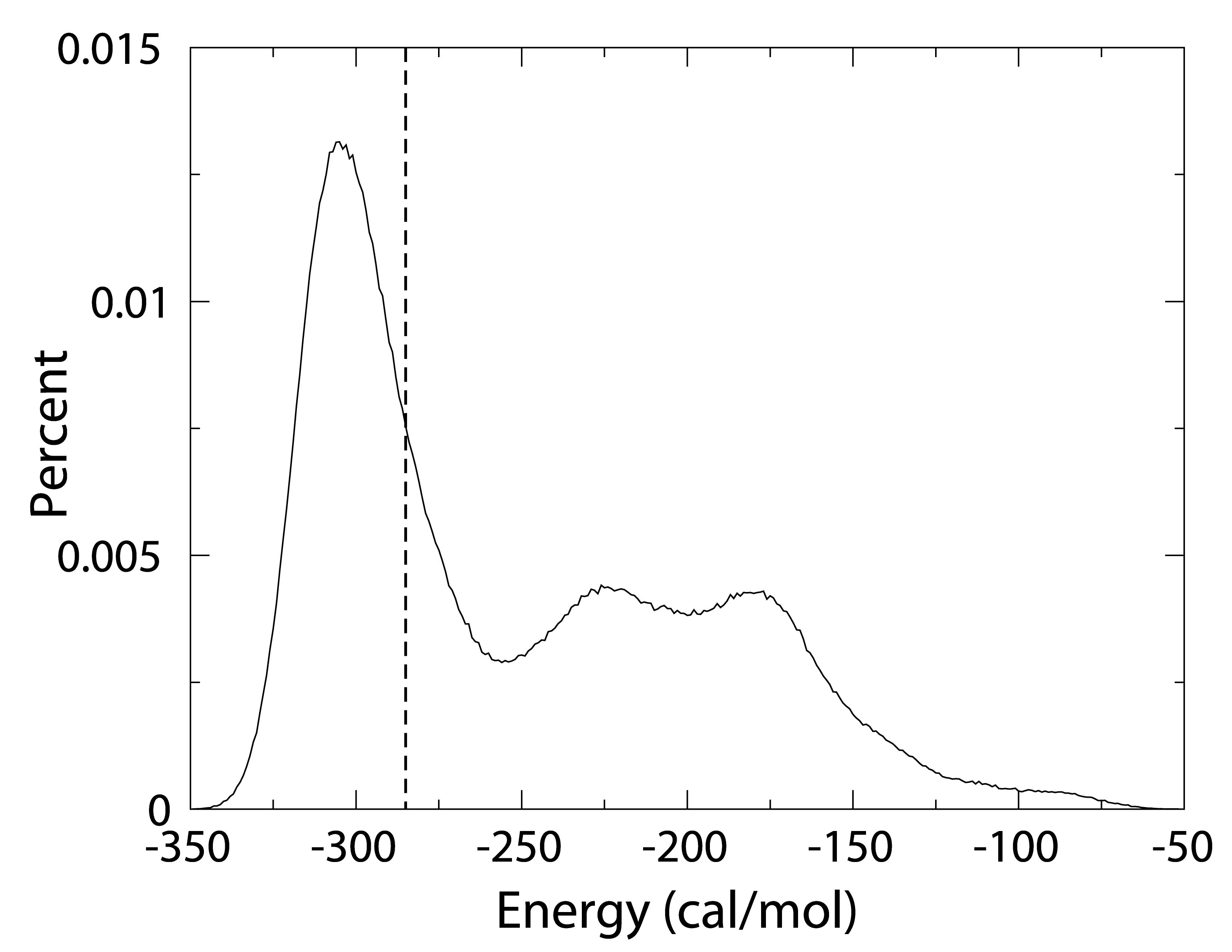

Supplement: Figure S2 — Example Energy Histogram. Example histogram of two replicas from one polypeptide (XN1Q23). The bin size is 1 cal/mol. The distribution for each replica is based on 1,000,000 data points: one value per time unit. Here, the data from two replicas are pooled; therefore this distribution is based on 2,000,000 data points. The dashed line indicates the energy cutoff value (−285 cal/mol); this cutoff applies to all replicas of this polypeptide. The cutoff is chosen such that only the lowest energy states are included as compact structures. (0.08 MB DOC) [file pcbi.1000772.s005.doc]
