# Supplementary material for: Polyglutamine Induced Misfolding of Huntingtin Exon1 is Modulated by the Flanking Sequences
Source: PLoS Comput Biol. 2010 Apr 29;6(4):e1000772. doi: 10.1371/journal.pcbi.1000772 (PMC2861695; doi:10.1371/journal.pcbi.1000772)

**Figure S3. Trajectories Sampling the Compact Domain.** Two example trajectories that depict how the simulations explored states that are compact and partially folded. Both trajectories are from the XN1Q_23_ simulation, and each trajectory corresponds to the particular replica indicated in the legend. The energy cutoff value is indicated as the red line. This value is determined by a histogram (Fig. S2). All explored states that occur below the cutoff are considered compact and are included in the compact ensemble.


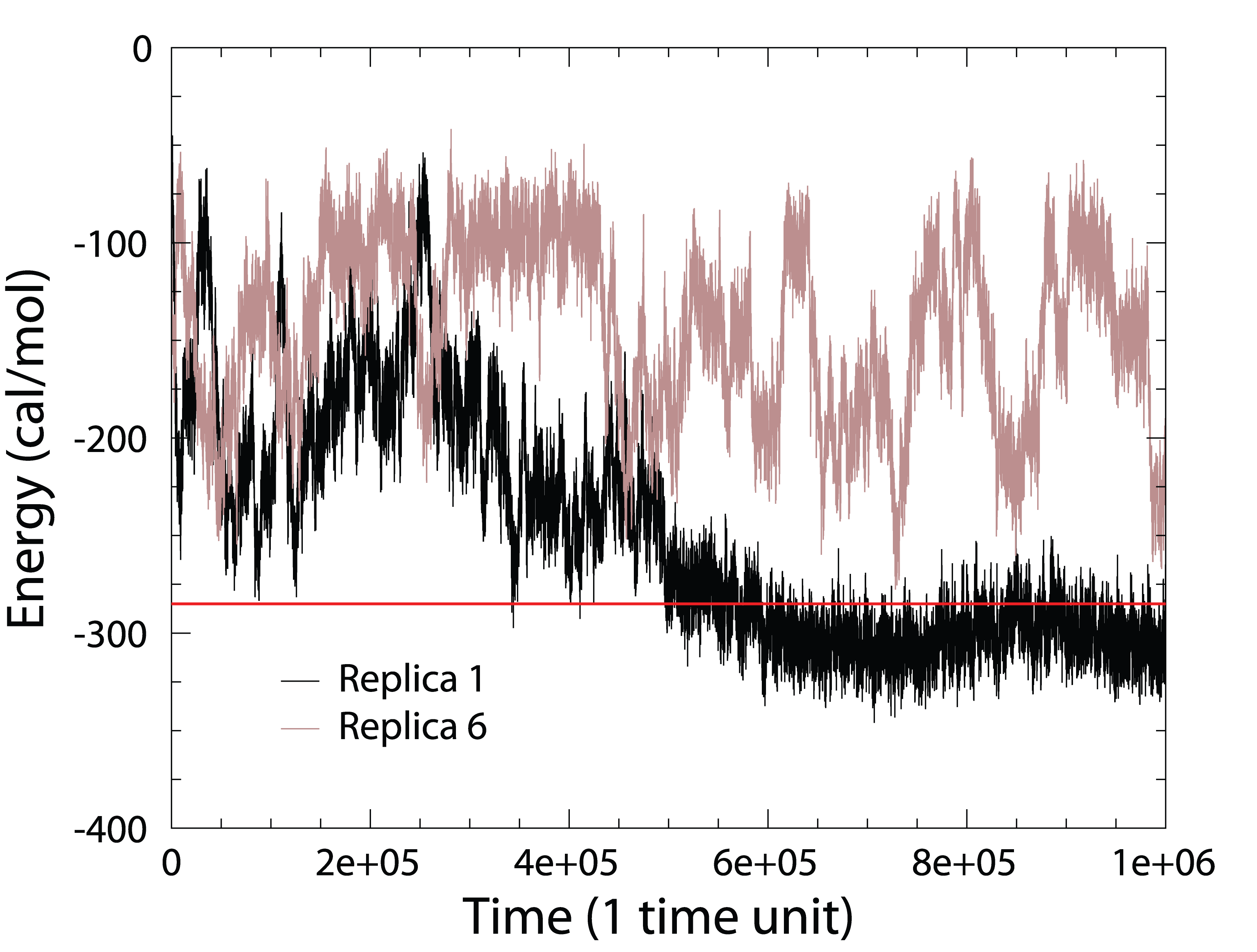

Supplement: Figure S3 — Trajectories Sampling the Compact Domain. Two example trajectories that depict how the simulations explored states that are compact and partially folded. Both trajectories are from the XN1Q23 simulation, and each trajectory corresponds to the particular replica indicated in the legend. The energy cutoff value is indicated as the red line. This value is determined by a histogram (Fig. S2). All explored states that occur below the cutoff are considered compact and are included in the compact ensemble. (0.13 MB DOC) [file pcbi.1000772.s006.doc]
