# Supplementary material for: Polyglutamine Induced Misfolding of Huntingtin Exon1 is Modulated by the Flanking Sequences
Source: PLoS Comput Biol. 2010 Apr 29;6(4):e1000772. doi: 10.1371/journal.pcbi.1000772 (PMC2861695; doi:10.1371/journal.pcbi.1000772)

**Figure S4. Hierarchical Clustering.** One example (from XN1Q_23_) of how the cutoff distance for clustering is determined. With hierarchical clustering, we are able to determine the number of clusters remaining for a given Root Mean Square Deviation (RMSD) cutoff.  The cutoff value, indicated by the dashed line (2Å), is chosen to maximize the clustering of similar structures while avoiding clustering of unlike structures. Clustering of similar structures occurs in the steeply descending portion of the curve (between 1Å and 2Å). In this region clusters are close to each other or similar, because a very small increase in the cutoff distance joins two clusters. Clustering of structures that are less similar occurs in the tail end of the curve (roughly 3Å or longer). Here, the clusters are distant or dissimilar and a large increase in the cutoff distance is required to join two clusters. Thus, a suitable cutoff is often found after the most similar clusters have been joined (here, between 1Å - 1.5Å) and before the distant clusters are joined (2.5Å and longer).


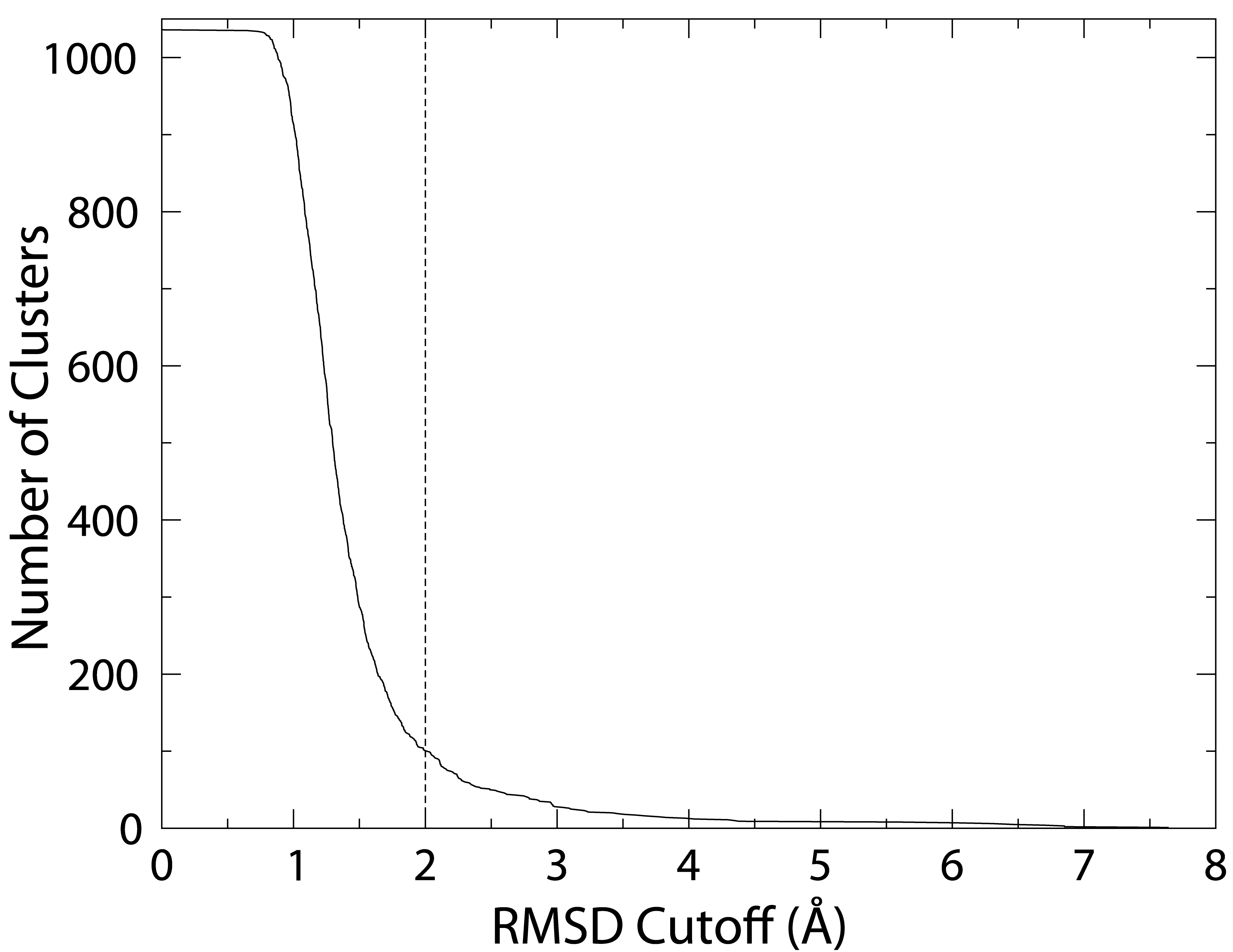

Supplement: Figure S4 — Hierarchical Clustering. One example (from XN1Q23) of how the cutoff distance for clustering is determined. With hierarchical clustering, we are able to determine the number of clusters remaining for a given Root Mean Square Deviation (RMSD) cutoff. The cutoff value, indicated by the dashed line (2Å), is chosen to maximize the clustering of similar structures while avoiding clustering of unlike structures. Clustering of similar structures occurs in the steeply descending portion of the curve (between 1Å and 2Å). In this region clusters are close to each other or similar, because a very small increase in the cutoff distance joins two clusters. Clustering of structures that are less similar occurs in the tail end of the curve (roughly 3Å or longer). Here, the clusters are distant or dissimilar and a large increase in the cutoff distance is required to join two clusters. Thus, a suitable cutoff is often found after the most similar clusters have been joined (here, between 1Å–1.5Å) and before the distant clusters are joined (2.5Å and longer). (0.08 MB DOC) [file pcbi.1000772.s007.doc]
